# “Many Things Grow in The Garden That Were Never Sown There”

**DOI:** 10.3201/eid2511.AC2511

**Published:** 2019-11

**Authors:** Byron Breedlove

**Affiliations:** Centers for Disease Control and Prevention, Atlanta, Georgia, USA

**Keywords:** art science connection, emerging infectious diseases, art and medicine, about the cover, Joaquin Sorolla y Bastida, Court of the Dances, Alcázar, Sevilla, Spain, many things grow in the garden that were never sown there, emerging pathogens, complex interactions, global climate change, human population growth and migration, antimicrobial resistance, agricultural practices

Among Europe’s oldest gardens are those at the Real Alcázar Palace in the heart of Seville, Spain. This palace and its gardens were designated as an UNESCO World Heritage Site in 1987. During 1910, King Alfonso XIII summoned artist Joaquin Sorolla y Bastida to Seville to paint a portrait of the king for the Hispanic Society of America. While waiting 3 weeks for the king’s arrival, Sorolla completed 18 paintings, including *Court of the Dances, Alcázar, Sevilla,* this issue’s cover art, one of a pair of paintings that shows different views of the same resplendent garden known for its fruit trees, produce, and fragrant flowers.

Sorolla scholars Blanca Pons-Sorolla and Monica Rodriguez Subirana describe Sorolla—frequently referred to as the “master of light” for his luminous, shimmering canvases―as “one of the most estimable, prolific, and fascinating personalities in the history of modern Spanish painting.” Although Sorolla is best known for “depicting the color, movement, and play of light on the sea like no one else,” Pons-Sorolla and Subirana point out that he also was one of the finest portraitists of his time and a noted landscape artist who “excelled at depicting gardens, one of the motifs he reproduced with the greatest splendor and sensibility.”

In *Court of the Dances, Alcázar, Sevilla*, Sorolla’s characteristically thick, dynamic brushstrokes capture the fecundity and textures of this sun-speckled garden. The intense blue sky glimpsed through the tangled vegetation contrasts with the deep green foliage. Spanish sunlight filters through tangles of flora, making leaves and fronds glow and shadows spill across the walkway, columns, and walls of this well-tended garden. Pons-Sorolla and Subirana note Sorolla “was interested in showing the sight lines around which the gardens were arranged, and how one entered these spaces through the leafy trees, placed in perspective with the background so as to convey a feeling of an infinite path.”

According to the J. Paul Getty Museum, which houses this work, “The courtyard of Seville's Alcázar Palace, the city's most splendid example of Moorish architecture, sparkles in the dappled summer sunlight. As always, Joaquín Sorolla y Bastida was concerned with color and light, brilliance and atmosphere. The colored reflections of the light animate the scene and help to define the forms, creating a sense that nature is ever-changing.”

“Many things grow in the garden that were never sown there” is among the enduring adages that English physician and writer Thomas Fuller included in his book *Gnomologia* (1732). That maxim, in its most literal sense, applies to various weeds, invasive plants, and other unwelcome flora that can surreptitiously infiltrate cultivated gardens such as those Sorolla depicted at the Alcázar Palace.

More broadly, this saying also provides a metaphor for the complex interactions of factors driving the surge in emerging infections across the globe, including climate change, human population growth and migration, antimicrobial resistance, and modern agricultural practices. Public health officials and researchers must be vigilant and focused in preparing for and responding to pathogens emerging in geographic locations and in populations where they were rare, nonexistent, or unknown.

**Figure:**
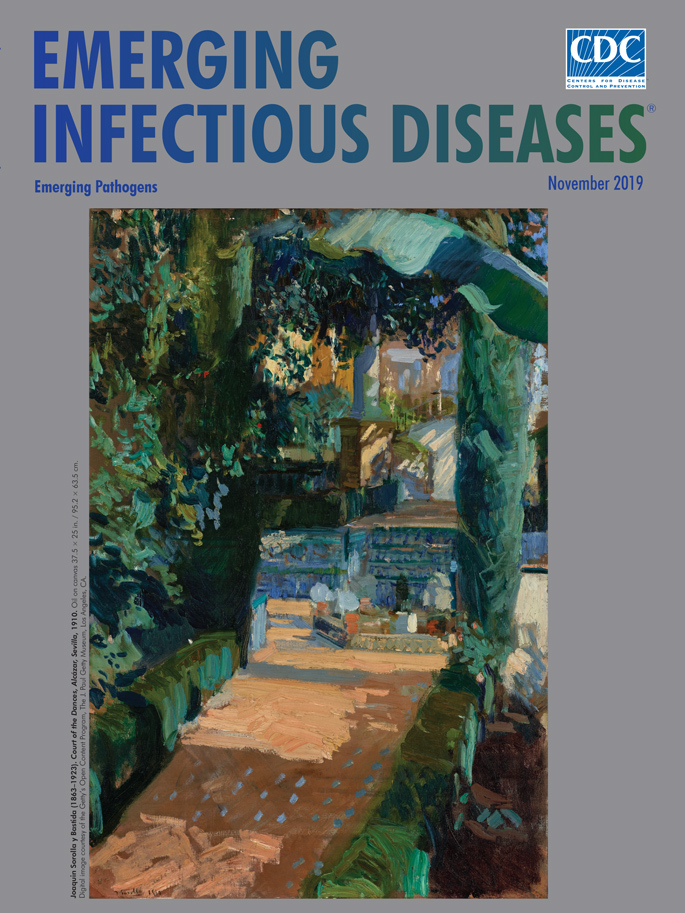
**Joaquin Sorolla y Bastida (1863–1923). Court of the Dances, Alcázar, Sevilla, 1910.** Oil on canvas 37.5 × 25 in./95.2 × 63.5 cm. Digital image courtesy of the Getty’s Open Content Program, The J. Paul Getty Museum, Los Angeles, CA, USA.
